# Adoptive T cell therapy: Addressing challenges in cancer immunotherapy

**DOI:** 10.1186/1479-5876-3-17

**Published:** 2005-04-28

**Authors:** Cassian Yee

**Affiliations:** 1 Fred Hutchinson Cancer Research Center, 1100 Fairview Ave N., D3-100, Seattle, WA 98109, USA

## Abstract

Adoptive T cell therapy involves the ex vivo selection and expansion of effector cells for the treatment of patients with cancer. In this review, the advantages and limitations of using antigen-specific T cells are discussed in counterpoint to vaccine strategies. Although vaccination strategies represent more readily available reagents, adoptive T cell therapy provides highly selected T cells of defined phenotype, specificity and function that may influence their biological behavior in vivo. Adoptive T cell therapy offers not only translational opportunities but also a means to address fundamental issues in the evolving field of cancer immunotherapy.

## Introduction

Augmentation of the immune response can be achieved through in vivo vaccination or ex vivo expansion of antigen-specific effectors followed by adoptive transfer. Both modalities share many features. For example, the antigen-presenting cell used for stimulating effector responses in vivo and in vitro represents a crucial element responsible for shaping the specificity and phenotype of the intended immune response. Therefore, preclinical studies that advance the engineering of robust antigen-presenting cells may be translated for use with either strategy. The cytokines necessary for augmentation and maintenance of the immune effector function and survival, the costimulatory factors required, and the regulatory and inhibitory mechanisms that must be overcome to achieve tumor eradication must be addressed whether vaccine strategies or adoptive T cell therapy is used. However, the behavior and ultimate fate of effectors generated in vivo can be substantially different from those generated in vitro. It would be naïve to assume that in vivo conditions could be reproduced completely by manipulating conditions in vitro and there may be effectors of desired phenotype and function that can only be generated or more easily generated in vivo than in vitro. On the other hand, when effectors can be generated in vitro, their specificity, magnitude, surface and functional phenotype can be far better defined than those generated following in vivo immunization. For this reason, the appeal of adoptive therapy is that the reasons for success or failure of a given strategy can be determined with greater precision than with in vivo vaccination. As more comprehensive and sensitive tools become available to monitor the immune response [1,2], this advantage may diminish; however it would be presumptuous to believe that immune monitoring can characterize induced vaccine-elicited effectors to the same degree as effectors elicited ex vivo. Although there is no guarantee that infused T cells will behave in vivo in the same manner as one would be able to predict in vitro, effector cells can be manipulated and selected ex vivo, prior to adoptive transfer, in a manner that can answer questions that cannot be addressed by vaccination strategies. When it is possible to generate an effector population of T cells of defined magnitude, the temptation however, is to ignore the role of qualitative differences by adopting a 'more is better' policy. The avidity, functional phenotype and in vivo 'survivability' are equally if not more relevant in mediating tumor eradication than numbers alone. With this in mind, the following commentary provides first a description of adoptive therapy strategies and then a point-by-point discussion of its various features and advantages in addressing challenges in immunotherapy.

### Defining Adoptive Cellular Therapy

Adoptive therapy involves the transfer of ex vivo expanded effector cells as a means of augmenting the anti-tumor immune response. Depending on the method of ex vivo selection, stimulation and expansion, varying degrees of uniformity with respect to antigen-specificity and phenotype may be obtained. This can range from a diverse polyclonal population of effector cells to highly selected T cell clones of defined phenotype, specificity and tumor avidity. The following broad and somewhat arbitrary categories describing T cell expansion methods are listed in order of increasing antigen specificity:

1. *Non-specific expansion of peripheral blood lymphocytes*. Non-specific ex vivo expansion of peripheral blood T cells by triggering the T cell receptor and costimulatory molecules with antibodies and/or the use of cytokines to drive T cells have been used in a number of clinical studies for the treatment of patients with HIV and malignant diseases [3-6]. In spite of the absence of a specific in vitro stimulator, in vitro studies suggest that augmentation of existing antigen-specific immunity can be achieved.

2. *Ex vivo expansion of Tumor infiltrating lymphocytes*. Expansion of infiltrating lymphocytes harvested from tumor sites yields a polyclonal population of T cells with broad reactivity to a variety of autologous tumor antigens. Although some degree of tumor-reactivity can be achieved, there is little control over the specificity or phenotype of the effector population without further in vitro manipulation [7]

3. In vitro stimulation to elicit antigen-specific T cells from peripheral blood lymphocytes using cells engineered for antigen presentation (peptide pulsed, RNA transfected or viral transduced autologous stimulator cells or artificial antigen presenting cells [8,9]) provides the most precise control over the specificity, and phenotype of the intended immune response [10,11]. Greater uniformity of effector specificity and phenotype may be achieved using clonal T cells expanded ex vivo.

Although this discussion is limited to the treatment of solid tumor malignancies, it should be noted that the adoptive transfer of allogeneic effector cells including donor lymphocyte infusions [12], minor antigen-specific CTL [13] and strategies that exploit NK alloreactivity [14] have been used successfully for the treatment of leukemia following allogeneic stem cell transplant. In addition, more refined approaches for the treatment of patients with post-transplant lymphoproliferative disease or Hodgkin's disease using EBV-specific T cells [15-17] have led to durable complete responses that have yet to be achieved with any notable degree in solid tumor immunotherapy. These studies are instructive for the solid tumor immunotherapist in that they demonstrate the feasibility of targeting specific tumor-associated viral antigens and provide insight into the role that T cells can play in eradicating life-threatening disease. Since solid tumors, such as melanoma, in its advanced stages exhibit physical and immunologic barriers distinct from tumors of hematologic origin, the following is limited to a discussion of immunotherapeutic strategies for the treatment of solid tumors.

Different strategies in generating effector cells for adoptive therapy influence not only the antigen-specificity, tumor avidity and cellular phenotype, but also the behavior of T cells in vivo i.e., longevity, trafficking, anti-tumor efficacy. Although effector cells generated using the above protocols can be considered in the following discussion, as a counterpoint to vaccination strategies, it will be assumed that the prototypic effector cells for adoptive therapy will be ex vivo expanded antigen-specific T cells.

### Antigen-specific Immunotherapy: Points to Consider

#### 1. Magnitude and Persistence

The magnitude of the anti-tumor immune response has been demonstrated in murine models of immunotherapy to be a critical factor in tumor eradication [18]. Although the frequency of antigen-specific T cells required to mediate an anti-tumor effect in patients is not known and is likely to vary widely depending on the antigen target, tumor burden, stromal environment and many other factors, including, perhaps most importantly, qualitative features of the effector cell. However, it may be agreed that there is a threshold below which, it would be unreasonable to predict a response, especially against established tumors, but it is equally unlikely that that threshold need be as great as 90% of all circulating CD8 T cells (unless such large numbers of cells are required to compensate for a paucity of desired effectors or a general qualitative defect). Tumor-eradicating therapy in murine models suggest that a frequency of antigen-specific T cells of at least 1–10% of CD8 T cells is required. In patients, this translates to a dose of 2 to 20 × 10^9 ^cells. The use of non-specific expansion methods (cytokines, TCR and costimulatory molecule triggering) have been used to successfully expand unselected peripheral blood mononuclear cells to > 10^10 ^cells in vitro over a period of 2–4 weeks [3,8]. In this case, however, the frequency of tumor-reactive T cells in the final infused product is often not known. Tumor-infiltrating lymphocytes after a 10–12 week period of in vitro culture with high-dose IL-2 yield 10^10 ^- 10^11 ^cells [7]; when expanded using anti-CD3 in combination with irradiated feeder cells, similar numbers can be achieved in less than half that time [19]. CD8 and CD4 T cell clones of defined antigen specificity and phenotype expand 500 to > 5000 fold over two weeks and can also achieve numbers > 10^10 ^[11,20-22]. In vivo, T cell frequencies of up to 5 % of all CD8 T cells in an unmanipulated host can be achieved. Patients have received adoptively transferred antigen-specific T cells numbering > 10^10 ^for a single infusion and often go on to receive multiple T cell infusions at intervals of days to weeks. In murine models, repeated infusions may play a role in 'attacking' the tumor in geographically distinct regions leading to tumor regression over time [23].

Equally important for successful therapy is the duration of in vivo persistence of transferred T cells which can vary from hours or days to weeks. This can depend in large part on the manner in which T cells were generated in vitro and under what conditions they are administered. Recent trials using adoptively transferred antigen-specific T cells are summarized in Table 1, Additional file 1 according to method of CTL generation and expansion, number of cells infused and immunomodulatory considerations such as prior lymphodepletion and dose of IL-2 administered. The methods for generating T cells varied with respect to the antigen presenting cell (*Drosophila *cells vs. autologous dendritic cells), in vitro dose of IL-2 ('T cell growth factor" to low-dose IL-2 at 10 U/ml to as much as 6000 U/ml), the number of cells infused and use of lymphodepleting regimens. In cases where polyclonal populations were used, as few as 10^8 ^antigen-specific CTL were infused; these T cells could not be detected in the peripheral blood (^a ^Mitchell et al, Table 1, Additional file 1) [24]. The absence of detectable T cells may be attributed to the absence of requisite costimulatory signals not provided by the gene-modified insect cells, absence of co-administered IL-2, the relatively low cell dose and/or underestimation of the actual frequency due to the use of limiting dilution analysis instead of tetramer staining. Although up to ten-fold higher doses of antigen – specific CTL clones were administered in other studies (^c ^Dudley et al) [25,26], these transferred cells also did not persist in vivo. In this case, T cells obtained from a previously vaccinated host failing peptide vaccine therapy, stimulated in vitro with the identical epitope and exposed to very high doses of IL-2 (^c&d ^Dudley et al)[25,26] are likely to behave very differently from T cells generated from a non-vaccinated host under more physiologic conditions of cyclical antigen-stimulation and low-dose cytokines (^b ^Meidenbauer et al; ^e ^Yee et al) [11,27,28]. In the former, adoptively transferred T cells experience a very short (< 48 hour) period of in vivo persistence possibly due to the requirement for supraphysiologic doses of IL-2 help in vivo and a starting population of T cells that may have reduced proliferative capacity due to prior in vivo vaccination. By contrast, T cells generated in vitro under more physiologic condition, can persist for more than 2 weeks in the presence of help (exogenous low-dose IL-2). Is this duration of in vivo persistence sufficient to mediate an anti-tumor response? While no clinical complete responses by RECIST (Response Evaluation Criteria In Solid Tumors) criteria [29] were noted in this study of patients with metastatic melanoma, what often escapes notice [30] is that patients experienced partial responses, significant tumor regression and stabilization of disease for an average of > 11 months and up to 29+ months – beyond what would be expected for patients with refractory disease following conventional therapy (median survival < 6 months) [11]. Since several of the patients who eventually progressed demonstrated evidence of outgrowth of antigen-loss tumor variants, it is suggested that such immunoselective pressure could not have occurred in the absence of effective antigen-specific immunotherapy [11].

One advantage of using ex vivo expanded T cells is that patients may have cells collected at an earlier stage of disease or prior to immunomodulatory therapy for later use. In one widely publicized study, TIL cells expanded using methods developed for clonal T cell expansion, were adoptively transferred to patients following lymphodepletion [19]. Melanoma-reactive T cells accumulated in the peripheral blood of these patients' reconstituting immune system to reach an astounding 97% of CD8+ T cells accompanied by tumor regression. Up to 50% of patients in an updated report demonstrated evidence of a clinical response and has been reported by its authors as a clear example of the role of adoptively transferred T cells, in the right setting to mediate dramatic clinical responses [31]. In corollary reports, the authors demonstrate that the level of T cell persistence observed in responding patients was significantly higher than that of nonresponding patients at early (5–15 days post infusion) and later timepoints (1–2 months post-infusion) suggesting that the duration of persistence of T cells derived from transferred, ex vivo expanded TIL play a role in the anti-tumor response [32]. Interestingly, although the infused product was comprised of several clonotypes, only a handful of T cell clones (among both responders and non-responders) persisted. This is an important study, but it leaves many questions unanswered and meaningful conclusions that advance the field are difficult to draw due to confounding variables in the design of this study. Without controlling for the specificity, dose or phenotype, it is unclear what type or dose of effector cells are required. Are CD4 T cells essential? What are the features of the subpopulation of T cells that experienced prolonged in vivo persistence and how do these differ from other T cells that were generated in vitro? Was their survival a result of greater (or lesser) avidity for their targets? Is it necessary to achieve the degree of lymphodepletion used in this study (which led to serious toxicities such as vision-threatening uveitis, and life-threatening PTLD)? Is selective depletion of regulatory cells or gentler preparative regimens to augment homeostatic mechanisms supportive of transferred T cells sufficient ? What role does high-dose IL-2 play in mediating clinical responses in this setting? Unfortunately, these answers cannot be divined from this study; rather, dissecting the contribution of these components to the anti-tumor response will be undertaken in carefully designed trials that exploit the advantages of using selectively expanded adoptively transferred T cells.

#### 2. Phenotype

The effector cell phenotype can be described as either 1) *surface markers *that are associated with specific (and non-exclusive) effector function – for example, CD4+ T cells are more likely to provide cytokine help than CD8+ T cells which are more likely to be cytolytic; or 2), a *functional *phenotype, such as tumor cell killing or TCR affinity. Vaccination strategies may be directed towards the induction of CD4 or CD8 T cells on the basis of whether Class I or Class II-restricted epitopes are used or whether the method of antigen engineering or presentation favors Class I or Class II MHC loading. However, more precise selection of the intended phenotype can be achieved by in vitro selection or enrichment of CD4 or CD8 T cells by immunomagnetic bead selection for example. T cells generated following peptide vaccination may exhibit low avidity for tumor cells, possibly as a result of preferential expansion of lower affinity effectors by APC presenting supraphysiologic concentrations of peptide MHC [2,33]. The use of altered peptide ligands may be capable of inducing in vivo an effector population with greater affinity for the tumor targets [34], but such ligands have not been frequently described and T cells of defined affinity cannot be selected. Perhaps part of the reason for the disparity between T cell frequency and clinical response in earlier vaccination studies is that attempts at immune monitoring enumerated T cells regardless of avidity. The study using the altered peptide ligand of CEA is one of only a handful of vaccine studies that demonstrated correlation with clinical response [34]. By contrast, T cells generated ex vivo with altered or natural peptide ligands or any other tumor-derived APC can be selected on the basis of the affinity of their TCR, overall tumor avidity or any other measurable and selectable functional property, uniformly expanded and transferred at a desired T cell dose [35-37].

One property of T cells that may be gauged by surface expression of specific markers, is their proliferative capacity, an important feature that will no doubt receive greater attention in the design of clinical trials, and paradoxically, may be inversely correlated with more routine measures of cytolytic or effector capacity (Gattinoni L et al, ISBTC abstract, 2004). In this aspect, although clones demonstrate significant proliferative capacity under the right conditions (e.g. IL-15 [38]), early effectors clearly exhibit greater potential for durable in vivo persistence. Perhaps studies demonstrating significant tumor responses [19] are a result of the presence of some of these early effectors in a polyclonal infusate of T cells or the adoptive transfer of smaller numbers of carefully selected effectors on the basis of proliferative capacity rather than tumor killing. Whether such an approach will be more successful, remains to be seen but represents the type of question that could best be addressed by adoptive immunotherapy.

#### 3. Specificity

As with vaccination strategies, the specificity of the intended immune response can be controlled and multivalent targeting can be achieved by adoptive therapy. However, eliciting responses by vaccination alone when the frequency of such responses is low as in the case of commonly shared tumor associated self antigens [39] or when such responses are represented by subdominant epitopes [40], may be limited by in vivo constraints. In this case ex vivo manipulation provides for a greater likelihood of generating T cells of desired specificity and magnitude by enriching for desired T cells in culture and careful selection of T cell clones. Alternatively, the ability to genetically modify T cells provides the opportunity to fashion T cells of defined specificity for adoptive therapy. T cell receptors cloned and sequenced from tumor-reactive lines and efficiently transferred into peripheral blood lymphocytes [41]. TCR-modified lymphocytes selected on the basis of in vitro markers or enriched by iterative stimulation have demonstrated the capacity to recognize and kill specific tumor target cells. In this way, patients for whom T cells of a given specificity are poorly represented by their immune repertoire, are not precluded from adoptive therapy when TCR-modified autologous lymphocytes can be used. Furthermore, mutant TCR can be designed to enhance affinity for the target MHC complex thus endowing genetically modified T cells with greater tumor avidity [42]. Finally, a chimeric T cell receptor comprised of an extracellular antibody binding a surface tumor target antigen coupled with intracellular signaling sequence (e.g. TCR-zeta) can also be used to endow peripheral blood lymphocytes with novel specificity [43,44].

#### 4. Genetic modification as a safeguard mechanism, to facilitate tracking and to enhance function

Other advantages associated with the use of genetically modified T cells for adoptive therapy over vaccination strategies are the capacity to eradicate T cells in vivo through the use of drug-inducible 'suicide' genes and to track these cells using genetic markers [45,46]. Although T cells transduced with early generations of the inducible HSV thymidine kinase gene were effectively eliminated in vivo following ganciclovir administration, they also suffered from early peripheral clearance due to the induction of an endogenous anti – HSV-TK response[47]. Later generations of suicide genes utilizing Fas-Fas dimerization technology address this problem and are being evaluated in pre-clinical studies [48]. T cell tracking with a unique genetic marker, such as a resistance gene for example permits unequivocal evaluation of T cell frequency, and localization if a feasible biopsy can be obtained from tumor or lymph node sites. Such cells can be analyzed using fluorescent-tagged riboprobes corresponding to the unique transgene or quantitative real-time PCR [45]. Dynamic T cell tracking in vivo, without the requirement for serial biopsies may be achieved using TK-transduced T cells that are designed to preferentially sequester radiolabelled substrate and can then be analyzed by PET imaging [49].

Genetic modification may also be used to enhance T cell function, for example, by conferring a helper-independent phenotype to antigen-specific CD8+ T cells with the use of a chimeric IL-2 receptor [50] or restoration of CD28 expression [51] enabling antigen-driven autocrine proliferation.

#### 5. Immune escape

Because T cells are isolated and expanded ex vivo, the clinical and immune state of the patient does not necessarily affect the ability to augment an immune response. Patients whose immune system may be crippled by tumor-suppressive factors or several rounds of chemotherapy and radiation may not be able to mount a robust immune response following vaccination. In these patients, ex vivo manipulation provides a means of isolating tumor-reactive T cells and expanding such cells for adoptive therapy. The mechanisms responsible for inhibiting an afferent response may limit the capacity of vaccines to generate functional T cells of sufficient magnitude. Ex vivo manipulation of T cells following exposure to immunomodulatory cytokines or selective depletion of regulatory cells (e.g. CD4, CD25+ T cells) may facilitate the isolation and expansion of tumor-reactive T cells for adoptive therapy. While it is possible to delete regulatory cells in vivo or co-administer immunomodulatory cytokines to augment a functional vaccine-elicited response [52,53], such strategies represent greater regulatory hurdles and can lead to unwanted effects. For example, the use of IL-2 to expand effector cells during the afferent phase of vaccine therapy can also lead to expansion of regulatory T cells in vivo [54] and depletion of regulatory T cells using anti-CD25 antibody can lead to the elimination of potentially beneficial CD25+ activated T cells.

#### 6. Feasibility considerations

The issue addressed here is the potential for adoptive T cell therapy to become a clinically significant modality that participates in the standard treatment of patients with malignant disease. The isolation and expansion of antigen-specific T cells is time and labor intensive, requires infrastructure support to cultivate and qualify T cell products and can be prohibitively expensive in its current experimental phase. Isolation and expansion of T cells for adoptive therapy can take 4–16 weeks and for patients with progressive disease, this may not be feasible. However, adoptive therapy can achieve T cell frequencies that are equal and often greater than that possible over the same period of time for patients receiving vaccines since in vivo expansion may also require several weeks and repeated boost administrations. Unlike vaccine reagents, T cell products cannot be manufactured and distributed easily; cryopreservation, storage, transport and reconstitution / thawing are problematic with a cell product. Vaccines can be made readily available in some forms to many institutions thus facilitating recruitment for the large-scale Phase II and Phase III studies needed to demonstrate efficacy and superiority over conventional modalities. In many ways, vaccination strategies have and are likely to gain regulatory approval more readily. Limitations to large-scale production of antigen-specific effectors ex vivo are being addressed. Most of these advances are related to adaptation of closed bag systems to eliminate the labor and inefficiencies of handling large numbers of cells, and to the design of artificial antigen-presenting cells to eliminate variability and reduce quality control concerns associated with in vitro cultured autologous APCs [9]. Advances in the isolation of antigen-specific T cells by cell sorting or immunomagnetic bead selection that can expedite the process, specialized reagents and culture vessels that facilitate expansion and storage and quality control measures that ensure product fidelity are currently being developed and will decrease many of these cost-related, and logistical issues.

## Conclusion

Our current understanding of the requirements for successful T cell-based therapy in the treatment of patients with solid tumors remains largely undeveloped Advances in this field will require judicious, step-wise translation of promising pre-clinical strategies into carefully designed clinical trials with discrete immunologic endpoints. This would be well-served by immunologic monitoring that encompasses not only a characterization of the biologic behavior of adoptively transferred or vaccine-elicited T cells in vivo but also a comprehensive analysis of immune escape mechanisms, especially those that develop with more and more effective strategies. Rather than claim clinical superiority of one modality over that of another on the basis of one or two early phase studies, it would be more instructive to exploit the individual advantages of vaccine or adoptive T cell therapy in designing clinical trials. Vaccine reagents can be easily produced and made readily available for widespread administration. This is particularly advantageous for later phase studies and multivalent approaches (especially where the antigen specificity is not known). Effectors may be more easily elicited in vivo; however, control over their desired features is less and the the burden for sophisticated immunologic monitoring much greater for vaccine strategies, where identification of T cells in vivo exhibiting such properties and correlating their presence with antitumor activity will be crucial. The implementation of adoptive therapy however belies its experimentalistic origins: in cases where a population of T cells of desired magnitude with defined phenotypic and functional properties is required, for example, to validate findings arising from vaccine studies or provide proof of principle for hypotheses based on pre-clinical studies, this represents the optimal strategy. In addition to extending exploratory research, there are translational opportunities afforded to the ability to manipulate effectors ex vivo that are otherwise not available to vaccines. In the end however, the difference in these two modalities can be considered largely arbitrary and there are complementary if not synergistic strategies utilizing both vaccination and adoptive T cell therapy [55] that will be essential for addressing challenges in cancer immunotherapy.

## Acknowledgements

CY is a Damon Runyon-Lilly clinical investigator supported (in part) by the Damon Runyon Cancer Research Foundation. This work was also supported by funding from R01 CA 104711, R21 CA 94500 and The Fialkav Award.

## Supplementary Material

Additional File 1 Recent trials using adoptively transferred antigen-specific T cells are summarized according to method of CTL generation and expansion, number of cells infused and immunomodulatory considerations such as prior lymphodepletion and dose of IL-2 administered. Click here for file
