# Supplementary material for: Adoptive T cell therapy: Addressing challenges in cancer immunotherapy
Source: J Transl Med. 2005 Apr 28;3:17. doi: 10.1186/1479-5876-3-17 (PMC1131930; doi:10.1186/1479-5876-3-17)
Supplement: Additional File 1 — Recent trials using adoptively transferred antigen-specific T cells are summarized according to method of CTL generation and expansion, number of cells infused and immunomodulatory considerations such as prior lymphodepletion and dose of IL-2 administered. [file 1479-5876-3-17-S1.rtf]

CTL generation	Cell Dose	Lympho-
depletion	IL-2	Other	Persistence	# Pts.	Trafficking to Tumor	Tumor response	Comments	
a Insect cells / PBMC + Tyr peptide 
+LD IL-2 (20U) /IL-7	~108 (1-2 x 10^8^ Tyr-specific) 
x 5 infusions
n=10 patients	_	_	_	< 24 h (LDA)	10	1 of 10 patients by 111In scan	PR (1 pt x 7 mths)
MR( 1 pt) 		
b DC + MART1 peptide
+ 1-2% "T cell growth    factor" 
x 4 cycles	~3.3 x 10^8^ 
MART1-specific x 3 infusions	_	LD
3 mU/d x 6d	_	2 –28 days 
(1.6 – 0.8%)	8	3 of 3 patients by 111In scan	PR (1)
MR (1)
SD (1 x 12 mths)		
c PBMC / TIL + G209M.
+ IL-2 (300U)
x 1 cycle -> clones
 	1.5 – 35 x 109
G209-specific
x 2-4 infusions
	_	IntD x 12 d

HD  x 4 d	(all pts failed G209M vaccine)

G209M 
post-inf. (3 pts)
	< 2 – 5 days	12	0 of 10 pts
by 111In scan	MR (2)	Both responses in HD-IL-2 arm	
d PBMC / TIL + G209M.
+ IL-2 (300U)
x 1 cycle -> clones
 	0.9 – 24 x 109
G209-specific
x 2 infusions
	Cytoxan + FLU	IntD x 12 d

HD  x 4 d	(all pts failed G209M vaccine)

G209M 
post-inf. (12 pts)	ND	12	ND	MR (5)	3 of 5 responses in HD-IL-2 arm	
e DC + MART1 or gp100 peptide 
+ IL-10 / IL-2 (10 U)
x 3-4 cycles -> clones	3.3 x 10^9^ / m2
MART-1 (5)
gp100 (5)-specific
    x 4 infusions	_	LD IL-2 x 14 d:
  1st    No IL-2
  2nd   250,000 
  3rd    500,000 
       4th   1,000,000 U/m2
	_	No IL-2:  <7 d
+ IL-2:    17 d	10	3 of 3 patients
by biopsy
confirmed by CDR3 seq.	Near CR
   (1, PET neg x 29 mths+) 
PR (1 x 8 mths)
MR (1)
SD (5 x 3-32 mths)	Antigen-loss tumor variants in 3 of 5 patients	
f TIL
+  IL-2 (6000 U/ml)	2.3 – 13.7 x 1010
MART1, gp100, unknown spec.
	Cytoxan + FLU	IntD x 12 d

HD  x 4 d	_	5 –63 d	13	2 of 2 patients by biopsy confirmed by Vbeta typing	Near CR (1, necrotic tumor)
PR  (5, 2-24 mths+)
MR (4)
	Updated study reports 18 of 35 responders	
a Mitchell MS et al J Clin Onc 20(4):1075. 2002
b Meidenbauer N et al J Immunol 170:2161. 2003
c Dudley ME et al J Immunother 24(4):363. 2001
	d Dudley ME et al J Immunother 25(3): 243. 2002
	e Yee C et al, PNAS 99(25): 16168. 2002
	f Dudley ME et al, Science 298:850. 2002 & Robbins PF et al, J Immunol 173:7125. 2004

TABLE 1.
